# Familial Hemiplegic Migraine and Spreading Depression

**Published:** 2014

**Authors:** Hadi KAZEMI, Erwin-Josef SPECKMANN, Ali GORJI

**Affiliations:** 1 Department of Pediatric, Shahed University, Tehran, Iran; 2 Shefa Neuroscience Research Center, Tehran, Iran; 3 Epilepsy Research Center, Westfälische Wilhelms-Universität Münster, Germany; 4 Institut für Physiologie I, Westfälische Wilhelms-Universität Münster, Germany; 5 Klinik und Poliklinik für Neurochirurgie, Westfälische Wilhelms-Universität Münster, Germany; 6 Department ofNeurology, Westfälische Wilhelms-Universität Münster, Germany

**Keywords:** Headache, Cerebrovascular diseases, Spreading depolarization, Children

## Abstract

**Objective:**

Familial hemiplegic migraine (FHM) is an autosomal dominantly inherited subtype of migraine with aura, characterized by transient neurological signs and symptoms. Typical hemiplegic migraine attacks start in the first or second decade of life. Some patients with FHM suffer from daily recurrent attacks since childhood. Results from extensive studies of cellular and animal models have indicated that gene mutations in FHM increase neuronal excitability and reduce the threshold for spreading depression (SD). SD is a transient wave of profound neuronal and glial depolarization that slowly propagates throughout the brain tissue and is characterized by a high amplitude negative DC shift. After induction of SD, S218L mutant mice exhibited neurological signs highly reminiscent of clinical attacks in FHM type 1 patients carrying this mutation. FHM1 with ataxia is attributable to specific mutations that differ from mutations that cause pure FHM1 and have peculiar consequences on cerebellar Cav2.1 currents that lead to profound Purkinje cell dysfunction and neuronal loss with atrophy. SD in juvenile rats produced neuronal injury and death. Hormonal factors involved in FHM affect SD initiation and propagation. The data identify SD as a possible target of treatment of FHM. In addition, FHM is a useful model to explore the mechanisms of more common types of migraine.

## Introduction

Hemiplegic migraine occurs both sporadically and as a familial syndrome. Familial hemiplegic migraine (FHM) is defined by migraine with aura and motor weakness and at least one first- or second-degree relative with migraine aura and motor weakness on one side of the body as a symptom. Sporadic hemiplegic migraine has the same clinical features as FHM, but with no family history of motor weakness (1).The ICHD-3 (beta version) diagnostic criteria for migraine with aura were changed during the 2004 and the 2013 editions. Now all attacks that meet criteria for migraines and include reversible weakness (motor aura) that last from 5 minutes to 72 h should be coded as hemiplegic migraine (familial or sporadic) once other causes, such as transient ischemic attack and stroke, have been excluded (2). In some patients, motor weakness may last weeks. Each individual non-motor aura symptom lasts 5–60 minutes and the aura is accompanied, or followed within 60minutes, by headache(2).Rarely, during FHM attacks, disturbances of consciousness(sometimes including coma), confusion, fever, and cerebrospinal fluid pleocytosis may occur.FHM attacks can be triggered by (mild to moderate) head trauma. In approximately 50% of FHM families, chronic progressive cerebellar ataxia occurs independently of migraine attacks. More than 60% of FHM patients also suffer from migraine attacks with typical aura or without aura, making FHM a useful genetic model to explore the mechanisms of more common types of migraine as well (3, 4).

FHM may be mistaken for epilepsy (postictal paresis, Jacksonian march, or Todd paralysis) and unsuccessfully treated. The duration of the gradual progression of the aura symptoms in FHM is 60 min on average, which is never seen in epilepsy, and that visual symptoms usually accompany paresis, which almost never happens in epilepsy (3).Inherited disorders associated with migraine headaches may include hemipares is such as CADASIL (5), MELAS (6), hereditary hemorrhagic telangiectasia (7), a form of hereditary amyloid angiopathy (8), familial cerebral cavernous malformation (9), and benign familial infantile convulsions (10).Magnetic resonance angiography revealed subtle narrowing of the left middle cerebral artery. The neuroimaging abnormalities completely resolved 24 h after attack onset. Multiple conventional and advanced MRI techniques play a key role in an FHM attack to exclude acute arterial ischemic stroke (11). 

On average 50% of children who have a parent with hemiplegic migraine will develop this disorder. The estimated prevalence of hemiplegic migraine is 0.01% (in the Danish population). Sporadic hemiplegic migraine and familial hemiplegic migraine have occurred with approximate equal prevalence. Hemiplegic attacks in familial hemiplegic migraine may begin as early as 5 to 7 years of age, with a mean age of onset of 12 years (range 1 to 51 years) in one study and 17 years (range 1 to 45 years) in another study (12, 13). Some patients with FHM have suffered from daily recurrent attacks since childhood (14). Hemiplegic migraine patients often stop experiencing attacks after the age of 50. Female-to-male gender ratios for hemiplegic migraine ranges from 2.5:1 to 4.3:1 (12, 15).Like other types of migraines, FHM affects women more than men.

FHM is an autosomal dominant, genetically heterogeneous disorder. Mutations in 3 genes are responsible for 50%–70% of published families with FHM.FHM is classified into FHM-1, FHM-2, and FHM-3, depending on specific genetic mutations. New genetic data have allowed for more precise data on FHM than was previously available. Specific genetic subtypes have been characterized as follows: 1. in FHM1 there are mutations in the CACNA1A gene (e.g., R192Q, S218L; coding for pore-forming α1A subunit of neuronal voltage-gated CaV2.1 channels)on chromosome 19p13; 2. in FHM2 there are mutations in the ATP1A2 gene (coding for a K/Na-ATPase) on chromosome 1q23; and 3. in FHM3 there are mutations in the SCN1A gene (coding for a sodium channel)on chromosome 2q24 (16). Mutations in ATP1A2 are associated with a rare form of hereditary FHM type 2 in some patients (17). There may be other loci not yet identified. Patients with type 1 mutations, which account for almost 50% of all FHM cases, have a 90% chance of experiencing a loss of motor function during a hemiplegic headache attack.FHM type 2 and type 3 mutations are much less common and are associated with recurrent seizures (18, 19).In experimental studies, FHM1 mutations shift calcium channel opening toward more negative membrane potentials and delay channel inactivation. Calcium channels open with smaller depolarization and stay open longer (20), presumably allowing more Ca2+ to enter presynaptic terminals that result in increased glutamate release to the extracellular compartment. This may be the mechanism of cortical and subcortical hyper excitability in patients suffering from FHM. It was suggested that altered P/Q-type calcium channel gating represents a common pathophysiological mechanism in approximately one-half of families. The same gene is also involved in the common forms of migraine with and without aura (21). A recent study showed that sibling pairs with any form of migraine, especially migraine with aura, had inherited the same 19p13 CACNA1A (22). The complex cellular and molecular environment in FHM could support a new tissue phenotype compatible with a neuroinflammatory profile. In patients with FHM, this condition might contribute to trigeminal pain pathophysiology through release of soluble mediators, including TNFα and may modulate the crosstalk between sensory neurons and resident glia, which is underlying the process of neuronal sensitization (23). Chemokine modulates neuronal activity and synaptic plasticity in neuronal tissue and may be involved in clinical symptoms such as headache and seizure attacks (24).

**The role of spreading depression (SD) in FHM**

SD is widely viewed as the electrophysiological event underlying migraine aura, and an intense self propagating front of neuroglial depolarization wave that spreads (2-3 mm/min) by way of gray matter contiguity, regardless of functional divisions (25). Underlying SD is a dramatic change in the distribution of ions between extra- and intracellular micromilieu. K+ and H+ release from the cells, while Na+, Ca2+ and Cl− enter together with water to the neurons causing cells to swell and the volume of the extracellular compartment to be reduced. SD is accompanied by an increase of glucose utilization and O2 consumption. Recovery of SD depends on energy metabolism (26, 27).

A brief period of neuronal excitation heralds SD, which is immediately followed by a prolonged nerve cell depression. A late excitatory period following the depression phase of SD occurred. It has been shown that induction of SD initially suppresses synaptic activity, which is followed by an irreversible potentiation of synaptic plasticity in human neocortical tissues obtained during epilepsy surgery(28, 29). Several investigations have also demonstrated a late excitable state of neuronal activities in various different brain structures remote from SD. Propagation of cortical spreading depression (CSD) is accompanied by the release and diffusion of several chemical mediators, such as excitatory amino acids, neurokinin, calcitonin gene-related peptide, serotonin, and brain-derived neurotrophic factors into the extracellular space, which may change the neuronal network excitability (26, 27).

SD belongs to the pathophysiology of the brain and there are reasons to believe that it is involved in several neurological disorders, including migraine with aura, cerebrovascular diseases (stroke, subarachnoid hemorrhage, intracerebral hemorrhage), head injury, epilepsy, and transient global amnesia (26, 30).

Available evidence strongly suggests involvement of SD in the pathophysiology of FHM type1. As mentioned, FHM type 1 is linked to chromosome 19p13 and mutations in the calcium channel α1A-subunit, CACNA1A, as well as to an altered P/Q-type calcium channel gating. Experiments on mutant mice provide mechanistic insight into modulation of SD susceptibility as a relevant mechanism in FHM. Mutations in the α1Asubunit of P/Q type calcium channels in mice attenuate evoked extracellular glutamate levels and increased the threshold for SD (20).The tottering mutation (a prolineto-leucine substitution in the S5-S6 linker region of the α1 subunit of the P/Q-type Ca2+ channel) causes loss-of-channel function, impaired presynaptic Ca2+ influx, and decreased neurotransmitter release that mainly inhibits excitatory neurotransmission (20, 31). 

Tottering mice show a ten-fold resistance to SD, with a slower SD propagation velocity and a failure to sustain regenerative propagation of the depolarization potential (32). Although these results indicate that mutations increased the SD threshold and are not consistent with the SD causative role in aura, they do reveal linkage between mutant P/Q type Ca2+ channels and SD (26). In contrast to this study, mutant mouse models expressing human FHM type 1 mutations in the same gene (R192Q or S218L) exhibit increased susceptibility to SD in the neocortex and the transient hemiplegia in response to SD initiation(20, 33, 34). The S218L variant exhibits larger gain-of-function (in vitro) and higher SD susceptibility (in vivo) in comparison to the R192Q mutation (33, 34). In contrast to pure hemiplegic migraine associated with the R192Q mutation, attacks in patients carrying the S218L mutation are sometimes accompanied by disturbances of consciousness, such as coma or stupor, and generalized seizures (18). 

Accordingly, neocortical SD induced pure hemiplegia in R192Q mutant mice, whereas S218L mutants additionally developed coma and often severe seizure attacks (33, 34).In addition, FHM type 1 mutant mice showed a facilitated subcortical propagation of cortical SD, which provide sa potential explanation for the hemiplegia, seizures, and coma in FHM patients(35). 

Further data suggest that sex hormones in addition to genetic factors modulate SD susceptibility in the animal model of FHM. Consistent with the female preponderance of FHM, female mice expressing the R192Q or S218L mutation showed a higher susceptibility than male mice toward SD (36). It has been shown that both estrogen and progesterone facilitate occurrence of SD in the neocortex and increase synaptic plasticity in these tissues (37). This may also affect the subcortical regions (38). This sex-related effect was completely reversed by an ovariectomy. Androgens appear to influence SD in the opposite direction in FHM type 1 mutant mice. Castration increased KCl-induced SD frequency and propagation speed. This was prevented by testosterone replacement in an androgen receptor-dependent manner (39).

It has been shown that induction of SD in the striate cortex modulates cerebellar photically evoked response. Knock-in mice that harbor human FHM1 mutations have a reduced threshold for SD and an increased probability of excitatory glutamate release (1). FHM1 with ataxia is attributable to specific mutations that differ from mutations that cause pure FHM1 and have peculiar consequences on cerebellar Cav2.1 currents that lead to profound Purkinje cell dysfunction and to neuronal loss with atrophy. Mental retardation might be diagnosed after the onset of severe FHM attacks (40). 

Severe attacks are suggested to result in permanent brain damage and atrophy as well as cognitive deterioration in the few children with normal early development and a notable stepwise developmental regression after the onset of severe FHM attacks. A decreased blood flow in the symptomatic cerebral hemisphere was reported in patients suffering from FHM (41). Small white matter lesions are not infrequent in children with migraine attacks (42). The potential fatal effects of repetitive cortical SD on the neurons of a juvenile brain have been reported (43). Repetitive cortical SD enhanced the production of dark neurons, reduced the mean volume of normal neurons, increased the number of apoptotic neurons, and enhanced expression of the NR2B subunit of NMDA receptors as well as the GluR1 subunit of AMPA receptors in the hippocampus and neocortex of juvenile rats(44).Using quantitative receptor autoradiography, an enhancement of glutamate N-methyl-d-aspartate (NMDA), α-amino-3-hydroxy-5-methyl-4-isoxazolepropionic acid (AMPA) and kainite receptor binding sites in somatosensory neocortical tissues was reported 1h after induction of CSD(45). The role of NMDA receptors in initiation of SD in human brain tissues is well-known (26). Intranasal application of the NMDA antagonist ketamine reversibly reduced the severity and duration of neurological deficit in FHM (46).Experimental models of CSD as a surrogate for aura can be useful in preclinical drug screening for migraine prophylaxis, including FHM, as well as for improving our knowledge of its basic mechanisms (47-50).

**In conclusion, **SD seems to play a crucial role in pathophysiology of FHM. Both genetic and hormonal factors in FHM affect SD initiation and propagation, and SD mimics symptom in animal models of FHM.

Therefore, SD may be a potential target for prevention of migraine attacks in FHM. Furthermore, FHM is a useful model to explore the mechanisms of more common types of migraine.

## References

[1] Russell MB, Ducros A (2011). Sporadic and familial hemiplegic migraine: pathophysiological mechanisms, clinical characteristics, diagnosis, and management. Lancet Neurol.

[2] The International Classification of Headache Disorders (2013). 3rd edition (beta version).Headache Classification Committee of the International Headache Society (IHS). Cephalalgia.

[3] Thomsen LL, Eriksen MK, Roemer SF, Andersen I, Olesen J, Russell MB A population-based study of familial hemiplegic migraine suggests revised diagnostic criteria. Brain 2002a.

[4] Kazemi H, Gorji A (2011). Migraine in children and adolescents. Iranian Journal of Child Neurology.

[5] Dichgans M, Mayer M, Uttner I, Brüning R, Müller-Höcker J, Rungger G (1998). The phenotypic spectrum of CADASIL: clinical findings in 102 cases. Ann Neurol.

[6] Prayson RA, Wang N (1998). Mitochondrial myopathy, encephalopathy, lactic acidosis, and strokelike episodes (MELAS) syndrome: an autopsy report. Arch Pathol Lab Med.

[7] Steele JG, Nath PU, Burn J, Porteous ME (1993). An association between migrainous aura and hereditary haemorrhagic telangiectasia. Headache.

[8] Haan J, Algra PR, Roos RA (1990). Hereditary cerebral hemorrhage with amyloidosis–Dutch type Clinical and computed tomographic analysis of 24 cases. Arch Neurol.

[9] Gunel M, Awad IA, Anson J, Lifton RP (1995). Mapping a gene causing cerebral cavernous malformation to 7q11.2-q21. ProcNatlAcadSci USA.

[10] Terwindt GM, Ophoff RA, Lindhout D, Haan J, Halley DJ, Sandkuijl LA (1997). Partial cosegregation of familial hemiplegic migraine and benign familial infantile epileptic syndrome. Epilepsia.

[11] Bosemani T, Burton VJ, Felling RJ, Leigh R, Oakley C, Poretti A, Huisman TA (2013). Pediatric hemiplegic migraine: Role of multiple MRI techniques in evaluation of reversible hypoperfusion. Cephalalgia.

[12] Ducros A, Denier C, Joutel A, Cecillon M, Lescoat C, Vahedi K (2001). The clinical spectrum of familial hemiplegic migraine associated with mutations in a neuronal calcium channel. N Engl J Med.

[13] Thomsen LL, Eriksen MK, Roemer SF An epidemiological survey of hemiplegic migraine. Cephalalgia 2002b.

[14] Vahedi K, Depienne C, Le Fort D, Riant F, Chaine P, Trouillard O (31). Elicited repetitive daily blindness: a new phenotype associated with hemiplegic migraine and SCN1A mutations. Neurology.

[15] Thomsen LL, Ostergaard E, Olesen J, Russell MB (2003). Evidence for a separate type of migraine with aura: sporadic hemiplegic migraine. Neurology.

[16] van den Maagdenberg AM, Haan J, Terwindt GM, Ferrari MD (2007). Migraine: gene mutations and functional consequences. Curr Opin Neurol.

[17] Gritz SM, Radcliffe RA (2013). Genetic effects of ATP1A2 in familial hemiplegic migraine type II and animal models. Hum Genomics.

[18] Ophoff RA, Terwindt GM, Vergouwe MN (1996). Familial hemiplegic migraine and episodic ataxia type-2 are caused by mutations in the Ca2+ channel gene CACNL1A4. Cell.

[19] Gargus JJ, Tournay A (2007). Novel Mutation Confirms Seizure Locus SCN1A is Also Familial Hemiplegic Migraine Locus FHM3. PediatrNeurol.

[20] Tottene A, Conti R, Fabbro A, Vecchia D, Shapovalova M, Santello M (2009). Enhanced excitatory transmission at cortical synapses as the basis for facilitated spreading depression in Ca(v)21 knockin migraine mice. Neuron.

[21] Terwindt GM, Ophoff RA, Haan J, Sandkuijl LA, Frants RR, Ferrari MD (1998). Migraine, ataxia and epilepsy: a challenging spectrum of genetically determined calcium channelopathies Dutch Migraine Genetics Research Group. Eur J Hum Genet.

[22] Terwindt GM, Ophoff RA, van Eijk R, Vergouwe MN, Haan J, Frants RR, Dutch Migraine Genetics Research Group (2001). Involvement of the CACNA1A gene containing region on 19p13 in migraine with and without aura. Neurology.

[23] Franceschini A, Vilotti S, Ferrari MD, van den Maagdenberg AM, Nistri A, Fabbretti E (2013). TNFα levels and macrophages expression reflect an inflammatory potential of trigeminal ganglia in a mouse model of familial hemiplegic migraine. PLoS One.

[24] Kodangattil JN, Möddel G, Müller, M, Weber W, Gorji A (2012). The inflammatory chemokine CXCL10 modulates synaptic plasticity and neuronal activity in the hippocampus. European Journal of Inflammation.

[25] Leao AAP (1944). Spreading depression of activity in the cerebral cortex. JNeurophysiol.

[26] Gorji A (2001). Spreading depression: a review of the clinical relevance. Brain Res Brain Res Rev.

[27] Somjen GG (2001). Mechanisms of spreading depression and hypoxic spreading depression-like depolarization. Physiol Rev.

[28] Berger M, Speckmann EJ, Pape HC, Gorji A (2008). Spreading depression enhances human neocortical excitability in vitro. Cephalalgia.

[29] Ghadiri MK, Kozian M, Ghaffarian N, Stummer W, Kazemi H, Speckmann EJ (2012). Sequential changes in neuronal activity in single neocortical neurons after spreading depression. Cephalalgia.

[30] Dreier JP, Major S, Pannek HW, Woitzik J, Scheel M (2012). COSBID study group. Spreading convulsions, spreading depolarization and epileptogenesis in human cerebral cortex.Brain.

[31] Tokuda S, Kuramoto T, Tanaka K, Kaneko S, Takeuchi IK, Sasa M (2007). The ataxic groggy rat has a missense mutation in the P/Q-type voltage-gated Ca2+ channel alpha1A subunit gene and exhibits absence seizures. Brain Res.

[32] Ayata C, Shimizu-Sasamata M, Lo EH, Noebels JL, Moskowitz MA (2000). Impaired neurotransmitter release and elevated threshold for cortical spreading depression in mice with mutations in the alpha1A subunit of P/Q type calcium channels. Neurosci.

[33] van den Maagdenberg AM, Pizzorusso T, Kaja S, Terpolilli N, Shapovalova M, Hoebeek FE (2010). High cortical spreading depression susceptibility and migraine-associated symptoms in Ca (v)21 S218L mice. Ann Neurol.

[34] Eikermann-Haerter K, Dileköz E, Kudo C, Savitz SI, Waeber C, Baum MJ (2009a). Genetic and hormonal factors modulate spreading depression and transient hemiparesis in mouse models of familial hemiplegic migraine type 1. J Clin Invest.

[35] Eikermann-Haerter K, Yuzawa I, Qin T, Wang Y, Baek K, Kim YR (2011). Enhanced subcortical spreading depression in familial hemiplegic migraine type 1 mutant mice. J Neurosci.

[36] Eikermann-Haerter K, Dileköz E, Kudo C, Savitz SI, Waeber C, Baum MJ (2009b). Genetic and hormonal factors modulate spreading depression and transient hemiparesis in mouse models of familial hemiplegic migraine type 1. J Clin Invest.

[37] Sachs M, Pape HC, Speckmann EJ, Gorji A (2007). The effect of estrogen and progesterone on spreading depression in rat neocortical tissues. Neurobiol Dis.

[38] Supcun B, Ghadiri MK, Zeraati M, Stummer W, Speckmann EJ, Gorji A (2012). The effects of titanic stimulation on plasticity of remote synapses in the hippocampus-perirhinal cortex-amygdala network. Synapse.

[39] Eikermann-Haerter K, Baum MJ, Ferrari MD, van den Maagdenberg AM, Moskowitz MA, Ayata C (2009c ). Androgenic suppression of spreading depression in familial hemiplegic migraine type 1 mutant mice. Ann Neurol.

[40] Freilinger T, Bohe M, Wegener B, Müller-Myhsok B, Dichgans M, Knoblauch H (2008). Expansion of the phenotypic spectrum of the CACNA1A T666M mutation: a family with familial hemiplegic migraine type 1, cerebellar atrophy and mental retardation. Cephalalgia.

[41] Crawford JS, Konkol RJ (1997). Familial hemiplegic migraine with crossed cerebellar diaschisis and unilateral meningeal enhancement. Headache.

[42] Candee MS, McCandless RT, Moore KR, Arrington CB, Minich LL, Bale JF Jr (2013). White Matter Lesions in Children and Adolescents With Migraine. Pediatr Neurol.

[43] Jafarian M, Rahimi S, Behnam F, Hosseini M, Haghir H, Sadeghzadeh B (2010). The effect of repetitive spreading depression on neuronal damage in juvenile rat brain. Neurosci.

[44] Sadeghian H, Jafarian M, Karimzadeh F, Kafami L, Kazemi H, Coulon P, Ghabaee M, Gorji A (2012). Neuronal death by repetitive cortical spreading depression in juvenile rat brain. Exp Neurol.

[45] Haghir H, Kovac S, Speckmann EJ, Zilles K, Gorji A (2009). Patterns of neurotransmitter receptor distributions following cortical spreading depression. Neurosci.

[46] Kaube H, Herzog J, Käufer T, Dichgans M, Diener HC (2000). Aura in some patients with familial hemiplegic migraine can be stopped by intranasal ketamine. Neurology.

[47] Ayata C, Jin H, Kudo C, Dalkara T, Moskowitz MA (2006). Suppression of cortical spreading depression in migraineprophylaxis. Ann Neurol.

[48] Kazemi H, Rahgozar M, Speckmann EJ, Gorji A (2012). Effect of cannabinoid receptor activation on spreading depression. Iran J Basic Med Sci.

[49] Seghatoleslam M, Ghadiri MK, Ghaffarian N, Speckmann EJ, Gorji A (2014). Cortical spreading depression modulates the caudate nucleus activity. Neuroscience.

[50] Eickhoff M, Kovac S, Shahabi P, Ghadiri MK, Dreier JP, Stummer W, Speckmann EJ, Pape HC, Gorji A (2014). Spreading depression triggers ictaform activity in partially disinhibited neuronal tissues. Exp Neurol.

